# The Bacterial Contamination of Allogeneic Bone and Emergence of Multidrug-Resistant Bacteria in Tissue Bank

**DOI:** 10.1155/2014/430581

**Published:** 2014-07-08

**Authors:** Fahmida Binte Atique, Md. Masudur Rahman Khalil

**Affiliations:** ^1^Department of Biochemistry and Microbiology, School of Life Sciences, North South University, Dhaka 1229, Bangladesh; ^2^Department of Microbiology, Gono Bishwabidyalay, Savar 1344, Bangladesh

## Abstract

Present study was carried out for the microbiological evaluation of allogeneic bone processed from femoral heads. A total 60 bacterial isolates comprising five different species including *Streptococcus* spp., *Staphylococcus* spp., *Klebsiella* spp., *Bacillus* spp., and *Pseudomonas* spp. were characterized based on their cultural and biochemical characteristics. Average bioburden was ranged from 5.7 × 10^1^ to 3.9 × 10^4^ cfu/gm. The majority (81.7%) of the microbial contaminants were detected as Gram positive with the predominant organism being skin commensal coagulase negative Staphylococci (43.3%). Antimicrobial resistance was evaluated by the activities of 14 broad and narrow spectrum antibiotic discs. Comparing the overall pattern, marked resistance was noted against Penicillin and Amoxicillin 100% (60/60). The most effective single antibiotics were Gentamicin, Tobramycin, and Ofloxacin which were bactericidal against 100% (60/60) isolates. Multidrug resistance (MDR) was confirmed in 70% (42/60) of the samples. Among them, the most prevalent antibiotypes were Penicillin, Amoxicillin, Oxacillin, Polymyxin, and Cefpodoxime (80% of total MDR). The study results revealed higher contamination rate on bone allografts and recommend the implementation of good tissue banking practices during tissue procurement, processing, and storage in order to minimize the chances of contamination.

## 1. Introduction

Human bone is the second most transplanted tissue after blood which has the unique ability to heal itself perfectly. It is estimated that more than 2.2 million bone grafting procedure annually take place worldwide in order to revise skeletal defects by replacement or augmentation [1]. In addition, bone grafts are also used to repair the defects in bone caused by birth defects, maxillofacial defects, traumatic injury, infections or enbloc resection of malignant tumours and in reinforcement of host bone prior to implantation of prosthesis [2–6].

Disease transmission and bacterial contamination are always a risk in allograft transplantation [7]. Thorough donor screening for the presence of transmissible diseases, bacterial testing, and aseptic processing practices can substantially reduce the risk but do not completely eliminate all the possible microbial contaminants from allograft [8]. So, for the safety of allogeneic tissue grafts, complete eradication of microorganisms is essential.

The risk of infectious disease transmission emphasizes the need of appropriate sterilization technique in tissue banking practice [9]. But the alteration in the biomechanical properties of particular tissues made it obvious that all forms of sterilization technique are not applicable [10]. Antibiotics has for long time been used to control infectious diseases. Even potentially fatal infections are now curable with the courses of antibiotics. But one of the alarming matters is that despite the development of new antibiotics with novel mechanism of action, it has become difficult to control the local bacterial prevalence and emergence of infectious diseases due to their resistance to the common antibiotics. Bacteria can defend themselves from the action of antibiotics by producing various metabolites which either degrade antibiotics or help bacteria to survive by various mechanisms.

## 2. Materials and Methods

### 2.1. Tissue Sample Collection

Tissue samples were collected through the donation of femoral heads removed during hip replacement, hemiarthroplasty, and traumatic limb amputation surgery from eleven hospitals of the Dhaka city including BDM Hospital, Center for Rehabilitation of Paralyzed Hospital, National Institute of Traumatology, Orthopaedic and Rehabilitation Hospital, Bangabandhu Sheikh Mujib Medical University Hospital, Ibn Sina Hospital, Bangladesh Medical College Hospital, Islami Bank Hospital, Central Hospital, Shikdar Medical college Hospital, and Al-Markajul Hospital and Trauma Center.

### 2.2. Tissue Donor Identification and Screening

All the tissues were collected by the written consent of the donor or next of kin by following “Human Organ/Tissue Donation and Transplantation Act” that has been passed by the National Parliament of the People's Republic of Bangladesh. The ages of donors were ranged from 40 to 75 years and all the donors were prescreened for the presence of transmissible diseases (e.g., HIV, HBV, and VDRL).

### 2.3. Initial Laboratory Processing and Bioburden Estimation

Fresh bones were collected under aseptic/sterile condition. During collection each container was labeled with donor ID and hospital registration number and kept at freezer (below −20°C). The plastic container with bone is placed in a cool box and transported immediately to the tissue banking laboratory. In the tissue banking laboratory the bones were preserved in freezer at −40°C. For the isolation, tissue samples were weighed by digital balance and taken into a sterile beaker containing 150 mL sterile normal saline and/or sterile distilled water. After using the orbital shaker the beaker containing the sample was gently shaken. 10 mL of suspension was taken by sterile pipette, which was sterilized by a sterilizer (at 180°C for 1 hour) into a test tube from the beaker. Then the sample was serially diluted up to 10^−4^. If discrete colonies were not detected in 10^−4^ dilution, further dilutions were prepared and the tests were then repeated. All the plates were incubated at 37°C for 24 hours. The bacterial colonies were counted after 24–72 hours.

### 2.4. Cultural Characterization and Biochemical Studies of Microbial Contaminants

The bacterial isolates, obtained from the selective and differential media, were characterized on the basis of their morphology (size, shape, and arrangement) by following Gram staining procedure. Cultural characteristics of the bacterial isolates were studied after 24–48 hours of incubation using freshly prepared reagents. According to Bargey's Manual of Determinative Bacteriology [11], several biochemical tests were performed to identify the biochemical characteristics of the bacterial isolates. The tests were Oxidase test, Catalase test, Indole production test, Methyl Red test, Voges-Proskauer test, Urease test, Citrate utilization test, Triple Sugar Iron test, and Carbohydrate (Lactose, Sucrose, and Dextrose) fermentation tests.

### 2.5. Antimicrobial Susceptibility Testing

Total 60 bacterial isolates were selected for antibiotic susceptibility test by Kirby-Bauer disc diffusion method described by Bauer et al. [12] using 14 broad and narrow spectrum antibiotic discs. Muller-Hinton agar plates were used to determine the antibiotic susceptibility of the bacterial isolates. A 0.5 McFarland was used as a standard tool to maintain the perfect turbidity. After swabbing with the bacterial suspension, antibiotic disks were placed aseptically over the inoculated media surface and at the same time spatial arrangement was maintained by means of sterile needle within a distance of 5 mm. Then the plates were incubated for 24 hours at 37°C. After the completion of incubation period, the plates were examined and the diameters of the clear zones were measured by a ruler in mm. The zone diameters were translated into susceptible (S), intermediate (I), and resistant (R) categories according to the National Committee for Clinical Laboratory Standards (NCCLS) [13].

## 3. Results

### 3.1. Determination of Bioburden in Bone

**Figure 1:**
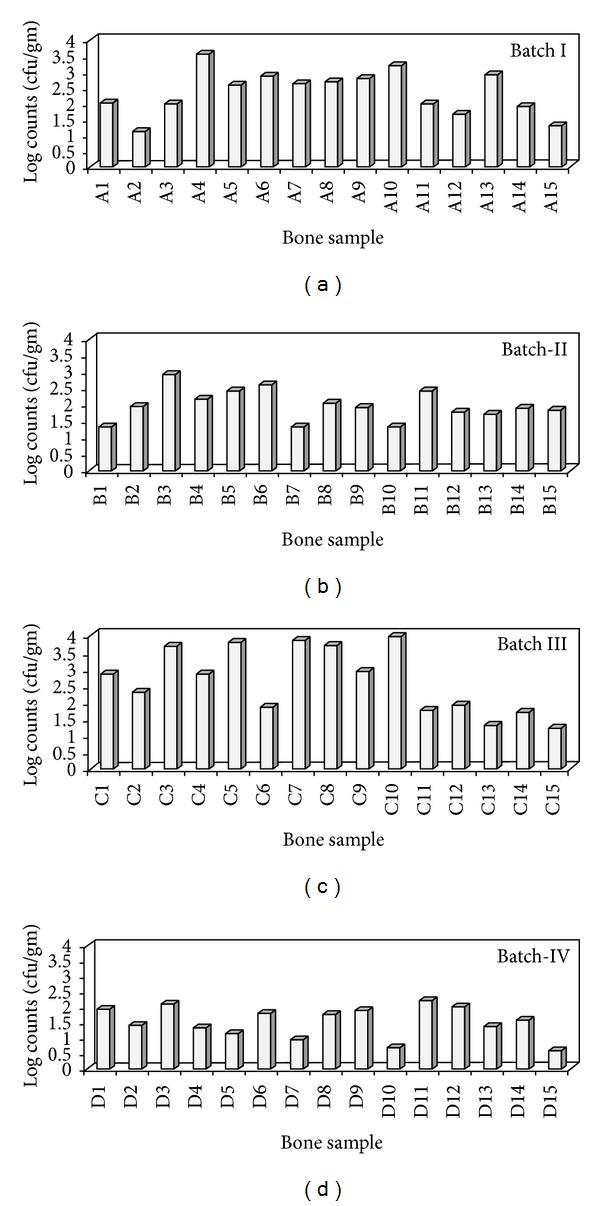
Microbial load of bone allografts from different batches of processing.

Microbial evaluation of bone allograft was carried out. A total 60 bacterial isolates obtained from 4 different batches of allograft processing. The bioburden varied from 0.57 to 3.94 Log cfu/gm. Maximum count was recorded for the first batch of processing, ranged from 3.23 to 3.94 Log cfu/gm. The lowest microbial levels from 0.93 to 1.92 Log cfu/gm were observed for the fourth batch. Microbial load of bone allografts from different batches of processing is presented in Figure 1.

### 3.2. Characterization of Bacterial Isolates

**Figure 2:**
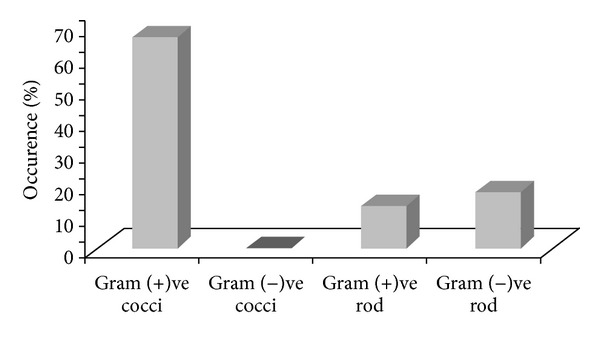
Types of microbial contaminants enumerated from bone.

Characterization of the bacterial isolates was performed based on their colony morphology. According to the Gram staining, majority (81.7%) of the microbial contaminants found as Gram positive, in which 67.8% were Gram positive cocci. The second most frequently isolated group was Gram positive bacilli as 13.9%. On the contrary, 18.3% of the microbial contaminants were Gram negative rods. No fungi or yeast were found. Types of microbial contaminants are presented in Figure 2.

### 3.3. Physiological and Biochemical Studies of the Bacterial Isolates

Several physicobiochemical tests were performed to identify the selected bacterial isolates up to genus level (Table 1). Based on the physiobiochemical characteristics, Twenty-one Gram positive cocci (B1, B5, B7, B14, B17, B19, B31, B32, B33, B34, B35, B39, B41, B42, B44, B45, B48, B50, B52, B58, and B59) were identified as* Staphylococcus* spp. and twelve Gram positive cocci (B3, B10, B11, B15, B21, B22, B26, B28, B29, B49, B53, and B55) were identified as* Streptococcus* spp. On the other hand, sixteen isolates of Gram positive rods (B2, B8, B16, B20, B25, B24, B30, B36, B38, B40, B43, B46, B51, B54, B60, and B63) were identified as* Bacillus *spp. Among the eleven Gram negative rods, eight of the bacterial isolates were* Pseudomonas* spp. (B4, B12, B18, B23, B27, B47, B56, and B57) and only three of the isolates were* Klebsiella* spp. (B6, B9, and B13).

### 3.4. Antibiogram Profile of the Bacterial Isolates

The bacterial isolates (*n* = 60) were subjected to antibiotic susceptibility test against 14 antibiotics from different groups including, Penicillin (P), Oxacillin (OX), Gentamicin (G), Erythromycin (E), Clindamycin (DA), Tobramycin (TOB), Ofloxacin (OXF), Polymyxin (PB), Azithromycin (AZM), Levofloxacin (LEV), Imipenem (IPM), Cefpodoxime (CPD), Amoxicillin (AML), and Meropenem (MEM) (Table 2). These antibiotics were selected upon the consideration of two facts: which antibiotics are the commonly prescribed by the physicians and which antibiotics are susceptible against bone contaminants. Disc diffusion method was used to frequently observe the antibiotic effects among the strains.

**Figure 3:**
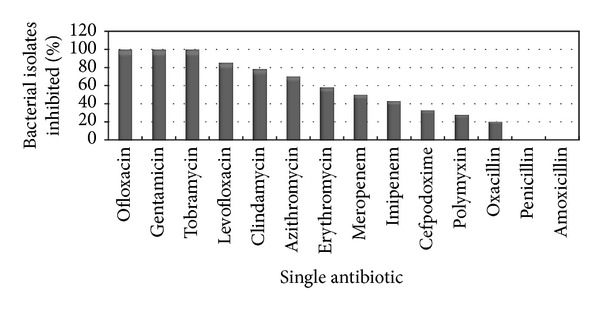
Percentages of antimicrobial resistance on bacterial isolates.

Among the 14 drugs, Penicillin and Amoxicillin were 100% (*n* = 60) resistant. On the contrary, Gentamicin, Tobramycin, and Ofloxacin were 100% sensitive. Apart from this, other drugs showed different level of resistance such as Oxacillin (80%), Polymyxin (70%), Cefpodoxime (60%), Imipenem (45%), Meropenem (40%), and Erythromycin (30%). Individual resistance and sensitivity pattern of the bacterial isolates is presented below (Figure 3).

Among the 60 bacterial isolates, 70% (*n* = 42) were multidrug resistant (MDR). The highest prevalent antibiotic resistance pattern was P, AML, OX, PB, CPD, IPM, E, MEM, and DA showed by bacterial isolates of batch-I. On the other hand, the lowest prevalent antibiotic resistance pattern was showed by batch-III as P, AML, OX, PB, and IPM (Table 3).

## 4. Discussion

The primary focus of our study was to determine the bioburden level of allogeneic bone. Study results showed that most of the samples were contaminated with Gram positive cocci specifically coagulase negative Staphylococci. Cultures were also positive for* Streptococcus* spp.,* Pseudomonas* spp.,* Bacillus *spp., and* Klebsiella *spp., respectively. The bacterial isolates found in our study are comparable with the previous reported studies. According to Saegeman et al. [14] in 36–38% cases infection of cadaveric bone and soft tissue allograft occurs due to coagulase negative Staphylococci especially that* Staphylococcus epidermidis* is the causative agent of disease. Deijkers et al. [15] analysed the bacterial contamination of bone allograft under aseptic operating condition and divided the organisms into low and high pathogenicity in which they considered organisms of low pathogenicity to be skin commensals and microorganisms of high pathogenicity were thought to be originated from endogenous sources in the donor, which more likely to cause infection in the recipient. Though* Streptococcus* spp. are not usually associated with graft infections, a survey study of tissue bank conducted by Vangsness et al. [16] reported about the invasive bacterial disease in which a 17-year-old male was found to be infected with* Streptococcus pyogenes* after reconstructive knee surgery. Ibrahim et al. [17] also reported that twelve of their bone allografts were contaminated with streptococci. Emergence of* Bacillus subtilis* and* Micrococcus* spp. was also summarized by many authors [18, 19]. Besides bacterial contaminations, environmental exposure, underlying diseases, and host defense mechanism can also contribute to the graft contamination in ratio between 2 and 5% [20].

We think that disease transmission can occur mainly in two ways: either through an infected donor or during tissue procurement, processing, even at the time of surgery in the operating theatre, as it has already been reported with surgical needles and suckers [21]. Bacterial transmission might be occurring from infected donor to recipient (tuberculosis and syphilis) or through viral transmission from infected donor (HIV and Hepatitis) or through bacterial contamination during procurement, processing, and storage of the bone allograft [22].

In order to avoid infection or diminish its incidences in bone allograft, strategies like careful donor selection, aseptic processing, proper use of disinfectants, and application of sterilization procedure with bacterial cultures need to be taken [23]. Even all the procedures are followed carefully, but what should be done if the culture from an implanted allograft is positive. The perioperative administration of systemic antibiotics is the choice to limit the infection which can occur after graft implant. This method is highly effective against bacteria while the effectiveness is depending on the constituents of antibiotics [24]. One of the feared complications is that, in our study, most of the bacterial isolates enumerated from bone showed multidrug resistance (more than one antibiotic) to the supplied antibiotics, as an explanation of such resistance might be the subsequent external contamination of the allograft. To prevent the endovascular graft infections, antibiotics are recommended to be used in the initial postoperative stage of bacterial seeding [25].

## 5. Conclusion

Bone allografts were found to be contaminated and about 80% of the contaminants were Gram positive. Study results also revealed the growing antimicrobial resistance of pathogens associated with the bone allografts. To minimize the contamination rate and to reduce the risk of dissemination of antibiotic resistant bacteria through the tissue allografts, it is suggested to use aseptic techniques in all the steps of allograft procurement, processing, and storage.

## Figures and Tables

**Table 1:** Summary of the biochemical tests of bacterial isolates.

Oxidase test	Motility test	IMViC test				TSI test			Suspected organism
		Indole	MR	VP	Citrate utilization	Slant	Butt	Gas production	
(−)ve	(−)ve	(−)ve	(+)ve	(+)ve	(−)ve	R	Y	(+)ve	*Staphylococcus *
(−)ve	(−)ve	(−)ve	(+)ve	(−)ve	(−)ve	R	Y	(+)ve	*Streptococcus *
(−)ve	(+)ve	(−)ve	(−)ve	(+)ve	(−)ve	R	Y	(+)ve	*Bacillus *
(+)ve	(+)ve	(−)ve	(−)ve	(−)ve	(+)ve	R	R	(−)ve	*Pseudomonas *
(−)ve	(−)ve	(−)ve	(−)ve	(+)ve	(+)ve	Y	Y	(+)ve	*Klebsiella *

**Table 2:** Antimicrobial susceptibility pattern of the bacterial isolates from bone allograft.

Antibiotics	Total number resistant/total number tested	% Resistance	Antibiotics	Total number resistant/total number tested	% Resistance
Oxacillin	48/60	80	Gentamicin	0/60	0
Imipenem	27/60	45	Polymyxin	42/60	70
Erythromycin	18/60	30	Ofloxacin	0/60	0
Penicillin	60/60	100	Meropenem	24/60	40
Clindamycin	6/60	10	Levofloxacin	6/60	10
Tobramycin	0/60	0	Azithromycin	12/60	20
Amoxicillin	60/60	100	Cefpodoxine	36/60	60

**Table 3:** MDR pattern of different bacterial isolates.

Total number of isolates	Resistance patterns	Multidrug resistance (MDR)
Batch-I (15)	P, AML, OX, PB, CPD, IPM, E, MEM, DA	(+)
Batch-II (15)	P, AML, OX, PB, CPD, E, AZM, LEV	(+)
Batch-III (15)	P, AML. OX, PB, IPM	(+)
Batch-IV (15)	P, AML, OX, PB, MEM, CPD, DA	(+)
